# Electrokinetic Power‐Series Solution in Narrow Cylindrical Capillaries for All Zeta Potentials

**DOI:** 10.1002/elps.202400183

**Published:** 2024-12-16

**Authors:** Sam Khalifa, Arturo Villegas, Francisco J. Diez

**Affiliations:** ^1^ Department of Mechanical & Aerospace Engineering, School of Engineering, Rutgers The State University of New Jersey Piscataway New Jersey USA; ^2^ SubUAS LLC Somerset New Jersey USA

## Abstract

Work from Rice and Whitehead showed the results of electrokinetic flow in a capillary tube under the assumption of low zeta potential <25 mV, limiting the approximation's usability. Further research conducted by Philip and Wooding provided an alternative solution that assumes high zeta potentials >25 mV and relies on Rice and Whitehead's solution for lower ranges. However, this solution is presented as a piecewise function, where the functions change based on the zeta potential and the κa parameter, introducing infinite values for the zeta potential and discontinuities in the derived functions. This paper aims to provide a singular equation solution to the full Poisson–Boltzmann equation for a long cylindrical capillary for all zeta potentials. This solution is a single, continuous, and finite function that produces exact results instead of approximations for all ranges of zeta potential. This exact solution is compared against published approximate solutions for large zeta potentials shown by comparing the large zeta potential approximation with the new exact solution. Important parameters such as volume transport and apparent viscosity were found to have errors of up to 9.76%–57.4%, respectively. The function $f(\kappa a,\psi_{0},\beta )$ has errors of up to 10.5% compared to our full solution.

## 1 Introduction

Electrokinetic flow is an important phenomenon used in various fields and has many applications. As an example, in the medical field, it is used for DNA sequencing, acts as an indicator for protein's stability, helps in understanding the interactions between cells and surfaces or between different cell types, and assesses the stability of colloidal suspension of nanoparticles in pharmaceutical drugs. It has also many uses in the fields of semiconductor processing, nanotechnology, water treatment and mineral processing, electrostatic coating, environmental science, and beyond.

The importance of this field has led to extensive research efforts since the early 1900s. The equation that describes the zeta potential for a cylindrical capillary, which was shown to be equivalent to a bed of fine particles or a porous diaphragm for large κa values [1], is formulated via the Poisson–Boltzmann equation for ions in the diffuse layer. For a single univalent solute, the differential equation takes the form of Equation (1).

(1) $$\psi^{\prime \prime }+\frac{\psi^{\prime }}{r}=\frac{8\pi ne}{\varepsilon }\sinh\frac{e\psi }{KT}.$$

Previous research conducted by Rice and Whitehead [2] used the approximation (2), which for room temperature water, is valid for zeta potentials of up to 25 mV.

(2) $$\sinh\frac{e\psi }{KT}\approx \frac{e\psi }{KT}.$$

Philip and Wooding [3] expanded on this work by formulating a solution for large values of the zeta potential. Their paper noted that the potential is increasing with respect to r. Therefore, even if the wall potential is higher than 25 mV, there could be some r such that zeta is smaller than 25 mV. They decided to have their solution be a piecewise function that is identical to Rice and Whitehead's solution when the potential is lower than 25 mV. For larger potentials, the solution is one of four functions [3, 4] that are all solutions to (1) using the approximation (3) when zeta is larger than 25 mV.

(3) $$\sinh\frac{e\psi }{KT}\approx \frac{\exp(\frac{e\psi }{KT})}{2}.$$

Both approximations use the following boundary conditions:

(4) $$\psi \left(a\right)=\psi_{0}\;;\;\frac{d\psi }{dr}{|}_{r=0}=0.$$

Rice and Whitehead's solution [2] famously yields the following function:

(5) $$\psi =\psi_{0}\frac{I_{0}\left(\kappa r\right)}{I_{0}\left(\kappa a\right)}.$$

The way Philip and Wooding's solution is constructed means that it is always a better approach since it will result in (5) when applicable. However, the different functions can make derivations difficult and the solution also leads to infinite values in the zeta potential [3, 4]. After Philip and Wooding published their results, Levine et al. expanded this work [4] by deriving some key functions from Philip and Wooding's solution and comparing them to Rice and Whitehead's, which serves as our main point of comparison for the paper. To this time, extensive research relies on Rice and Whitehead's solution due to its simplicity such as [5, 6, 7, 8]. While some newer papers require more accurate results and use Philip and Wooding's solution such as [9, 10, 11, 12].

To avoid working with multiple functions, validate the results in [3], and obtain accurate results in future research, a full solution is necessary. The lack of a full solution so far resulted in researchers needing to run computational fluid dynamics simulations such as in [13, 14, 15, 16, 17]. One could also use symbolic operations to obtain a single numerical result like in [18, 19].

## 2 Methodology

We start by normalizing (1) as done in [3] to get

(6) $$\frac{1}{R}\frac{d}{dR}\left(R\frac{d\Psi }{dR}\right)=\sinh\Psi ,$$

where R=κr and $\Psi =\frac{e\psi }{KT}$. Since there is only one regular singularity about R=0 when we expand the left‐hand side, we assume that the potential function is a power series with a radius of convergence that is infinite and we obtain

(7) $$\Psi \left(R\right)=\sum_{i=0}^{\infty }a_{i}R^{i}.$$

We then focus on the left‐hand side of (6). By taking the *n*th derivative, we obtain

(8) $$\frac{d^{n}}{dR^{n}}\left(\frac{1}{R}\frac{d}{dR}\left(R\frac{d\Psi }{dR}\right)\right).$$

By taking the normalized zeta function to be the power series and then substituting it for the derivative, we obtain

(9) $$\frac{d^{n}}{dR^{n}}(\frac{1}{R}\frac{d}{dR}\left(R\sum_{i=1}^{\infty }ia_{i}R^{i-1}\right).$$

Multiplying by R and then taking the derivative once more, we obtain

(10) $$\frac{d^{n}}{dR^{n}}\left(\frac{1}{R}\sum_{i=2}^{\infty }i^{2}a_{i}R^{i-1}\right).$$

Now dividing by R and taking the derivative, we arrive at the following:

(11) $$\sum_{i=2}^{\infty }i^{2}\frac{(i-2)!}{(i-2-n)!}a_{i}R^{i-2-n}.$$

This can be evaluated at R=0, which will be used later to obtain $a_{i}$ given $a_{i-2}$

(12) $$LHS\left(0\right)=a_{n+2}{\left(n+2\right)}^{2}n!$$

We assign a series for both sinhΨ and coshΨ as follows:

(13) $$\sinh\Psi =\sum_{i=0}^{\infty }b_{i}R^{i},$$

(14) $$\cosh\Psi =\sum_{i=0}^{\infty }c_{i}R^{i}.$$

If we perform series multiplication and set similar power coefficients to be equal, we obtain the following:

(15) $$b_{i}=\frac{1}{i}\sum_{k=0}^{i-1}\left(k+1\right)a_{k+1}c_{i-1-k},$$

(16) $$c_{i}=\frac{1}{i}\sum_{k=0}^{i-1}\left(k+1\right)a_{k+1}b_{i-1-k},$$

Equations (15) and (16) allow us to find $b_{i}$ and $c_{i}$ in terms of all the previous a, b, and c coefficients as well as $a_{i}$. If we set $a_{0}$ such that it provides a wall zeta potential as in (4), we also obtain $b_{0}=\sinh a_{0}$ and $c_{0}=\cosh a_{0}$. Taking the *n*th derivative of (6) while evaluating at 0 and utilizing (12), we arrive at

(17) $$a_{n+2}{\left(n+2\right)}^{2}n!=b_{n}n!$$

Which can be simplified to

(18) $$a_{n+2}=\frac{b_{n}}{{\left(n+2\right)}^{2}}.$$

Here $a_{i}$ is the coefficient in the power series in (7), which will be denoted as $a_{i,\Psi }$ to highlight that it is for the normalized zeta potential, Ψ, the first six nonvanishing terms are given in terms of $a_{0,\Psi }$ in the Appendix. Similarly, we will use $a_{i,\psi }$ for the nonnormalized zeta potential ψ. Given the definition of the normalized R and Ψ, we can see that for the nonnormalized equation, the coefficients are obtained by (19).

(19) $$a_{i,\psi }=\frac{KT}{e}\kappa^{i}a_{i,\Psi }.$$

**FIGURE 1:**
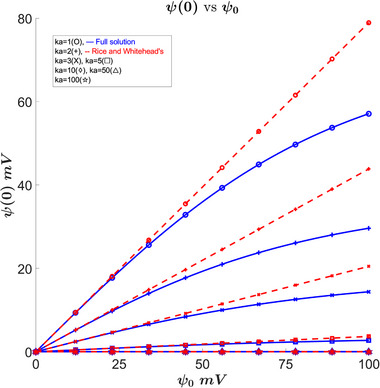
Comparison of the zeta potential at the center versus zeta potential at the wall for our full solution and Rice's approximation. Results show considerably smaller central potential than the approximation as the wall zeta potential becomes larger than 25 mV.

In Figure 1, we can see how the potential at the center of the capillary changes with $\psi_{0}$ and κa, compared to Rice's approximation via plugging r=0 in Equation (5). The central potential $a_{0}$ is a common parameter for a nonlinear solution. For example, in capillary slits, the full analytical solution exists but relies on the central potential and any function derived from the zeta potential is defined in terms of the central potential [20]. This is due to the boundary conditions for (1) being defined at the center and the wall. So we must know both the wall potential as well as the the central potential. In Figure 1, we can see that compared to Rice's approximation, the central potential is lower, this gives us a higher bound on our iterative search for $a_{0}$.

## 3 Results and Discussion

### 3.1 Solution Validation

**FIGURE 2:**
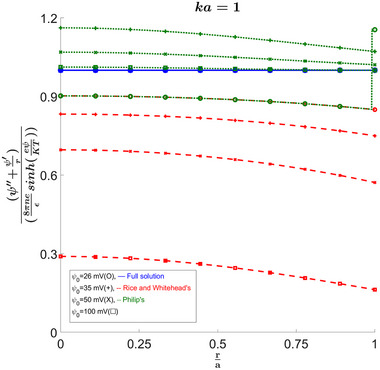
Validity of our full solution to the differential equation and comparison to the approximated solutions from previous work. The figure shows how the full solution is accurate for all zeta potential ranges, while the approximations fail to accurately verify the equation except for zetas much larger or much smaller than 25 mV. Values used for the constants are given in the Appendix.

For illustration purposes and without loss of generality for all the results displayed in the following figures, we set the parameter $\beta^{*}=0.25$, where $\beta =\beta^{*}\Psi_{0}^{2}=\beta^{*}{\left(\frac{e\psi_{0}}{KT}\right)}^{2}$. The parameter β is defined as in [2], and $\beta^{*}$ is defined as in [4]. The number of terms used here was such that the ratio on the *y*‐axis of Figure 2 was 0.99..9 with 16 repeated nines at the wall where it is the furthest from 1. This was determined to be sufficient for convergence since most values derived from the wall potential seem to converge at around a minimum of 0.9 and Rice and Whitehead's solution had a value of 0.9997 for $\psi_{0}=1\;\text{mV}$, where the solution is known to be valid.

The improvement of the full solution can be shown for κa=1, where we can see in Figure 2 that our full solution remains at 1 for all zeta potentials at the wall, while for Rice's approximation we see that the value deviates further from 1 as we increase $\psi_{0}$ as expected. Philip and Wooding's solution seems to be more accurate as $\psi_{0}$ increases. This shows that the solution is indeed accurate and that we can use it to calculate important values related to the flow and compare them to the two approximations. Since the solution is in series form, taking derivatives and integrals becomes trivial, and therefore finding properties such as the velocity profile or volume transfer becomes much simpler.

### 3.2 Electro‐Osmotic Volume Transfer Under No Pressure Gradient

**FIGURE 3:**
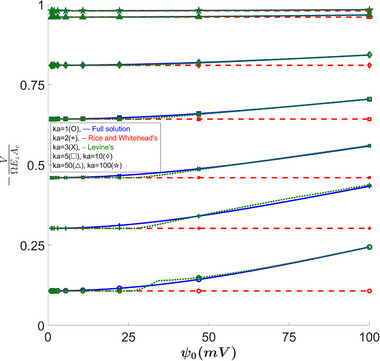
Electro‐osmotic volume transfer versus $\psi_{0}$. Comparing our full solution to Levine's results shows noticeable errors in the 25–35 mV zeta potential range. Rice's solution shows very large errors for high wall potentials at small κa values.

In Figure 3, as well as all of the following plots, we can see that when κa becomes very large, this is, the Debye length becomes very small compared to the capillary, then all of the solutions converge to the Smoluchowski result [21]. We also see that for a κa value of 2, both solutions slightly underestimate the volume transfer for a larger zeta potential range. For κa=1, we see the largest error of 9.76% at $\psi_{0}=26\;\text{mV}$, which does not vary with β.

### 3.3 Evaluating Figure of Merit Function *f*($\kappa a,\psi_{0},\beta$)

An important and useful function that appears when evaluating properties' ratios is *f*($\kappa a,\psi_{0},\beta$). In [2], this function simplifies down and is no longer a function of $\psi_{0}$; nevertheless, this function is defined such that the following equations are satisfied at their given condition:

(20) $$f\left(\kappa a,\psi_{0},\beta \right)=\frac{\psi_{a}}{\psi_{0}}={\left(\frac{\lambda }{\Omega }\frac{E_{z}}{P_{z}}\right)}_{i=0}={\left(-\frac{\lambda }{\Omega }\frac{V}{i}\right)}_{P_{z}=0}.$$

**FIGURE 4:**
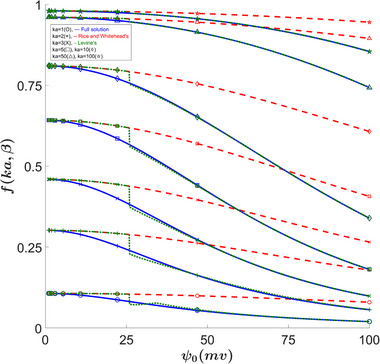
*f*($\kappa a,\psi_{0},\beta$) versus $\psi_{0}$. Comparing our full solution to Levine's results shows noticeable errors in the 15–25 mV zeta potential range for low κa values. Rice's solution shows very large errors for high wall potentials at all κa values.

**FIGURE 5:**
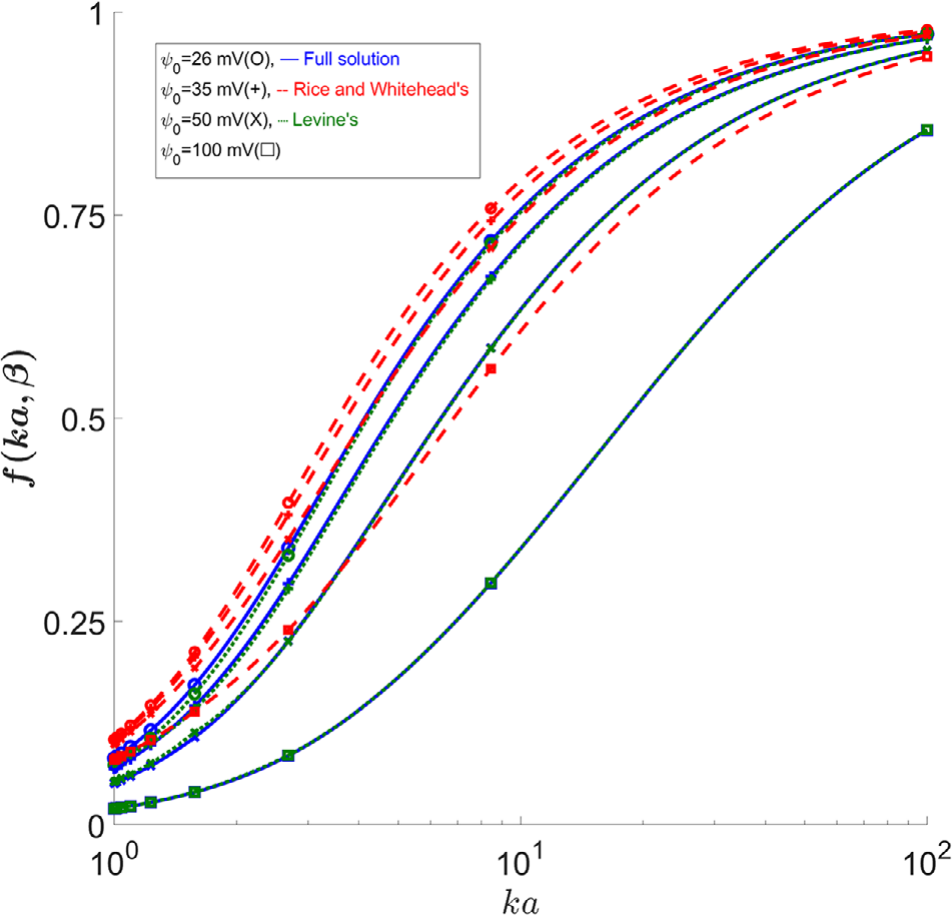
*f*($\kappa a,\psi_{0},\beta$) versus κa. The figure highlights the discrepancy between our full solution and Levine's from Rice's even for large κa values.

Although the function f does not depend on $\psi_{0}$ for a low zeta approximation, the fact that we set $\beta^{*}=0.25$ means that with a changing $\psi_{0}$, β will also change, resulting in a nonconstant f graph for Rice's approximation. As shown in Figure 4, we see the same area where the largest discrepancy occurs. However, a κa of 2 becomes accurate again for $\psi_{0}>40\;\text{mV}$. We also notice that the error grows with κa initially until around a κa of 10, where the error starts to decrease again. The reason why Figures 4 and 5 show such a high error for Rice's solution is due to an approximation made in the conduction current where it was assumed $i_{2}=\lambda E_{z}A_{c}$ [2], while our full solution as well as Levine's results uses the full equation which becomes important as we look at larger values for $\psi_{0}$. Here, we use the following equation [4]:

$$i_{2}=2\pi \lambda E_{z}\int_{0}^{a}r\cosh\left(\frac{e\psi }{KT}\right)\;dr.$$

This results in large errors in Rice's solution even when κa is large. For κa=1, we see the largest error of −10.5% at $\psi_{0}=35\;\text{mV}$, at a value of β=0.1, which is applicable for high‐temperature flows as well as larger capillaries.

### 3.4 Apparent Viscosity

**FIGURE 6:**
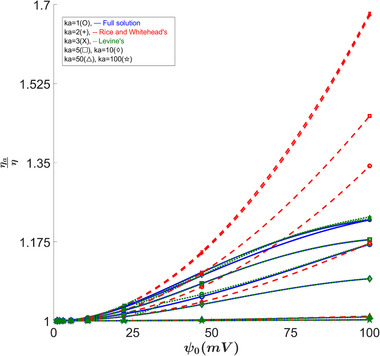
Apparent viscosity ratio versus $\psi_{0}$. Rice's solution diverges due to approximation errors, while the full solution shows small but consistent errors from Levine's results for κa= 2.

**FIGURE 7:**
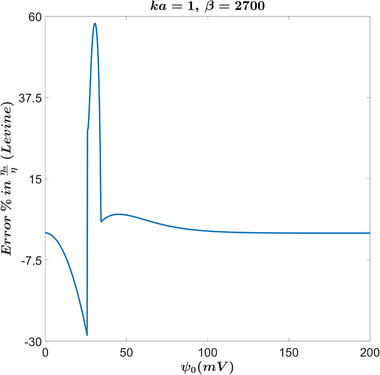
Percent error in $\frac{\eta_{a}}{\eta }$ versus $\psi_{0}$. This figure highlights extremely large errors in Levine's results.

**FIGURE 8:**
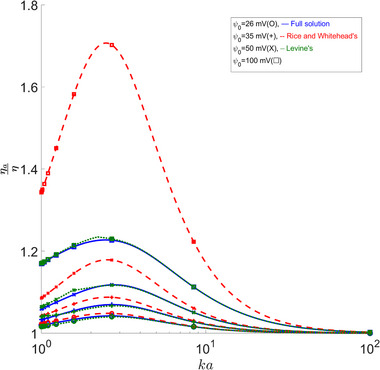
Apparent viscosity ratio versus κa. Comparing our full solution to other solutions shows noticeable errors in the 1–2 κa range for zeta = 50 mV and the 1.5–2.5 κa range for zeta = 100 mV.

Another property that highlights the difference between the full solution and the approximations that was also studied in [2, 4] is the apparent viscosity. From Figures 6 and 8, we can see an overestimation of the apparent viscosity for Rice's approximation. For Levine's results, we see that they are in better agreement. However, there is still a meaningful discrepancy for lower κa values, especially for larger β values, which are applicable for smaller capillaries or at lower temperatures, where we reach errors of about 57.4% at 31mV as seen in Figure 7. For a more standard value of β of 27 [2], we still see errors of around −23% at 25mV.

All high κa values seem to agree as seen in Figures 6 and 8.

### 3.5 Radial Dependence and Discontinuities

**FIGURE 9:**
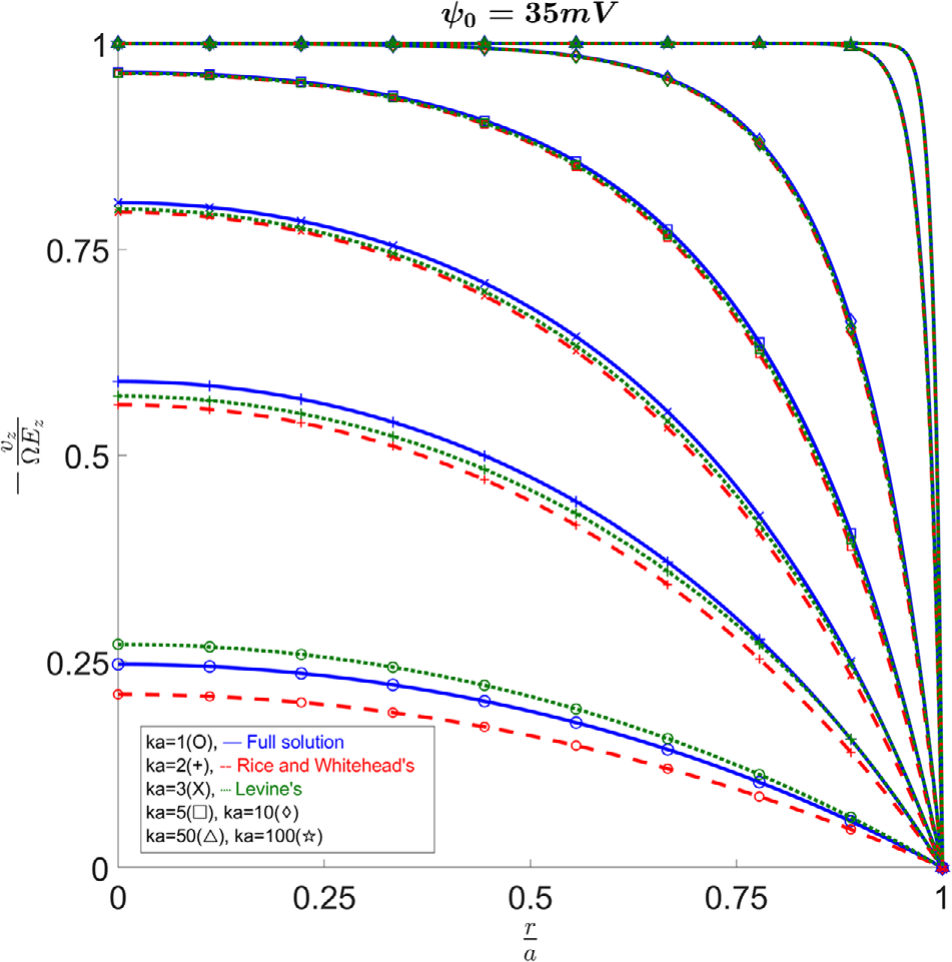
Electro‐osmotic velocity versus radius at $\psi_{0}=35\;\text{mV}$. Comparing the full solution to Levine's results shows overestimation for κa= 1 and underestimation for κa = 2 and appropriate approximation for κa ≥ 3.

**FIGURE 10:**
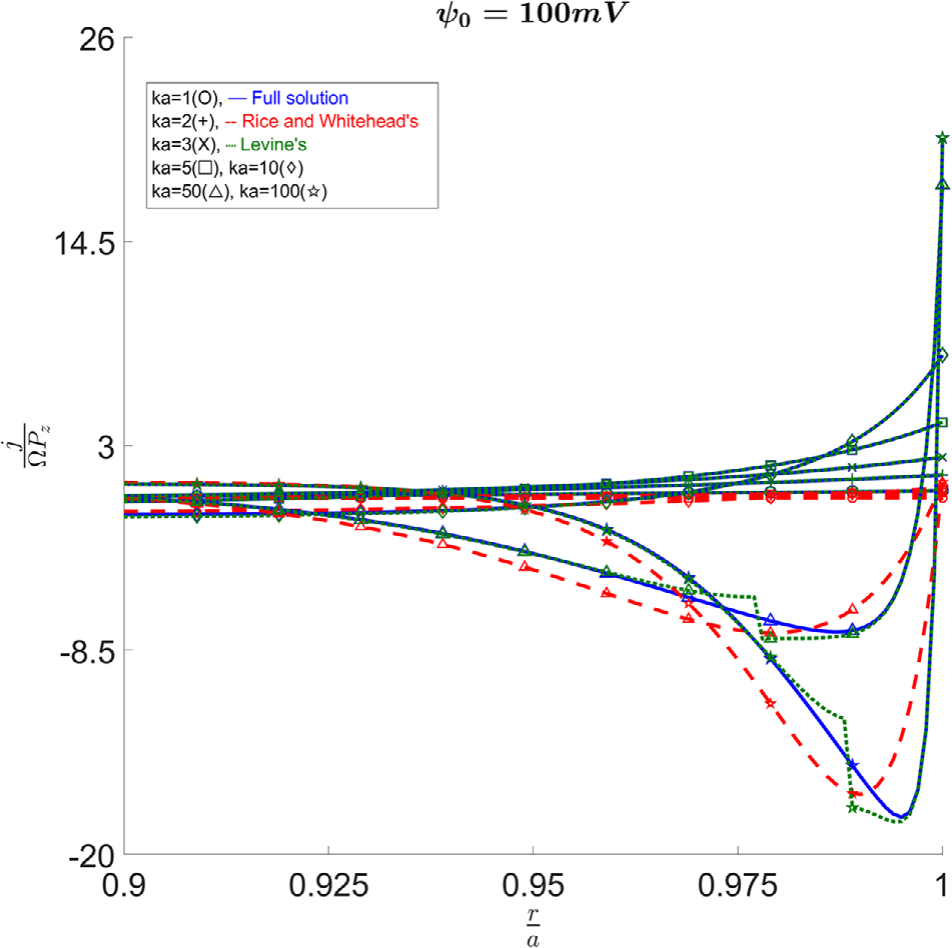
Current density versus radius at $\psi_{0}=100\;\text{mV}$. Comparison shows large errors for Rice's solution and large discontinuous jumps in Levine's results.

Figure 9 shows the variation between the different solutions and their predictions for electro‐osmotic velocity. Again, as κa increases, all the solutions converge; however, we can see that for lower κa values they disagree. For a κa of 1, we see that Philip and Wooding's solution results in an overestimation, while for κa values higher than 2, it results in an underestimation. Additionally, Figure 10 shows us that if an important understanding of the behavior across the capillary is desired, Levine's results do not suffice. Levine et al. [4] as well as Philip and Woodings [3] talk in more detail about why these discontinuities and infinite values arise. It is worth noticing that, away from the discontinuity point, Levine's results quickly converge to our full solution on both sides of the discontinuity. We also observe how the approximation matches in other plots such as Figure 8. Therefore, it seems that the deviations on both sides cancel out in the integrals to produce similar results when looking at the flow across the capillary instead of at a particular point.

## 4 Conclusion

This paper proposes and validates a full power‐series analytical solution to the Poisson–Boltzmann equation of the electrokinetic flow in long cylindrical capillaries. This is the first time a complete, continuous solution has been derived for this equation without relying on specific approximations. We also note that, in Kang et all (2002) [9], a full solution to the transient electrokinetic flow inside a capillary was developed. However, this solution needed to integrate a function that contained the zeta potential and their solution relied on the approximation in [3]. Unlike previous simplified solutions, our full solution is valid for all zeta potentials, filling the gap between low and high wall potentials. We also observed the largest differences to Levine's approximated solution for low values of the κa parameter, with large errors between 20 and 35 mV. The largest errors were found in the viscosity estimate, where classical results underestimate the viscosity by up to 57.4%. Future work will focus on applying this solution for multiple solutes and nonunivalent solutes and comparing the results from [9] for transient time using the same method with our full solution instead of the approximation in [3].

## Nomenclature

$A_{c}$
: cross‐sectional area of capillary
a
: radius of the capillary
$a_{i}$
: coefficient of $R^{i}$ in polynomial for Ψ
$b_{i}$
: coefficient of $R^{i}$ in polynomial for sinhΨ
$c_{i}$
: coefficient of $R^{i}$ in polynomial for coshΨ
$E_{z}$
: axial electric field gradient
e
: elementary charge of an electron
$f(\kappa a,\psi_{0},\beta )$
: $\frac{\psi_{a}}{\psi_{0}},\;\frac{\lambda }{\Omega }{\frac{E_{z}}{P_{z}}}_{i=0},\;-\frac{\lambda }{\Omega }{\frac{V}{i}}_{P_{z}=0}$
i
: electric current through the capillary
j
: electric current density
K
: Boltzmann's constant
n
: ions per unit volume
$P_{z}$
: axial pressure gradient
R
: normalized radius inside capillary
r
: radius inside capillary
T
: absolute temperature
V
: volume transport
β
: $\frac{\Omega^{2}\eta \kappa^{2}}{\lambda }$
ε
: dielectric constant of fluid
η
: fluid viscosity
$\eta_{a}$
: apparent fluid viscosity
κ
: reciprocal of Debye length
λ
: fluid conductivity
Ω
: $\frac{\varepsilon \psi_{0}}{4\pi \eta }$
Ψ
: normalized zeta potential
ψ(0)
: zeta potential at the center of the capillary
ψ
: zeta potential
$\psi_{0}$
: zeta potential at the wall
$\psi_{a}$
: apparent zeta potential at the wall

## Conflicts of Interest

The authors declare no conflicts of interest.

## Data Availability

Data sharing is not applicable to this article as the work was based on theory, therefore no datasets were generated or analyzed during the current study.

## APPENDIX A

The constants used in Figure 2 are a=100nm, $\kappa =\frac{1}{a}=10^{7}\;m^{-1}$, $e=1.6*10^{-19}\;C$, ε=81, T=300K, $K=1.381*10^{-23}\;\frac{J}{K}$, and $n=\frac{\varepsilon KT\kappa^{2}}{e^{2}8\pi }$. n is defined in terms of κ as in [2] so that it does not need to be redefined when we change the value of κa.[Table elps8082-tbl-0001]

**TABLE A1 elps8082-tbl-0001:** The first six nonvanishing terms for $a_{i,\Psi }$.

$a_{0,\Psi }$	$a_{0}$
$a_{2,\Psi }$	$\frac{\sinh\left(2a_{0}\right)}{8\cosh\left(a_{0}\right)}$
$a_{4,\Psi }$	$\frac{\sinh\left(2a_{0}\right)}{128}$
$a_{6,\Psi }$	$\frac{3\sinh\left(4a_{0}\right)-2\sinh\left(2a_{0}\right)}{18432\cosh\left(a_{0}\right)}$
$a_{8,\Psi }$	$\frac{9\sinh\left(4a_{0}\right)-16\sinh\left(2a_{0}\right)}{589824}$
$a_{10,\Psi }$	$\frac{135\sinh\left(6a_{0}\right)-165\sinh\left(4a_{0}\right)-63\sinh\left(2a_{0}\right)}{353894400\cosh\left(a_{0}\right)}$
